# 2-Year clinical outcomes following combined iliotibial band and gluteus medius percutaneous ultrasound tenotomy in refractory GTPS

**DOI:** 10.1016/j.inpm.2025.100717

**Published:** 2025-12-04

**Authors:** Ugur Yener, Mohamed Awad, Tahereh Naeimi, Kyle Max Mayblum, Sebastian H. Lujan Varela, Joseph M. Seldin, Hatice Begum Ciftci, Alaa Abd-Elsayed, Sayed Emal Wahezi

**Affiliations:** a Department of Physical Medicine & Rehabilitation, Montefiore Medical Center, Bronx, NY, USA; b White Plains Hospital, White Plains, NY, USA; c Scarsdale Medical Group, Harrison, NY, USA; d Department of Physical Medicine and Rehabilitation Jacobi Medical Center, NY, USA; e Department of Anesthesiology, University of Wisconsin, Madison, WI, USA

## Abstract

**Background:**

Greater Trochanteric Pain Syndrome (GTPS) is a common cause of chronic lateral hip pain, often refractory to conservative medical management (CMM). Combined percutaneous ultrasound-guided tenotomy (PUT) of the gluteus medius (GMed) and iliotibial band (ITB) has shown promising one-year results, but data on long-term outcomes remain limited. This study evaluates two-year clinical outcomes in patients undergoing combined GMed and ITB PUT for recalcitrant GTPS.

**Methods:**

This retrospective cohort study included 69 patients (79 hips) treated with combined GMed and ITB PUT between January 2022 and August 2023. Baseline and follow-up data, collected for an average of 2-year, were obtained through chart review and structured phone interviews. The primary outcome was ≥50 % reduction in Numeric Rating Scale (NRS) pain scores. Secondary outcomes included side-lying tolerance, sitting-to-standing ability, responder rate and need for additional hip interventions. Responder status required both pain reduction and complete side-lying tolerance. Statistical significance was evaluated using paired t-tests with p < 0.05.

**Results:**

Median baseline Numeric Rating Scale (NRS) pain score was 10 interquartile range (IQR 9–10), decreasing significantly to 2 (IQR 1–4) at 1, 6, and 12 months, then increasing to 6 (IQR 4–10) at 24 months (p < 0.001 vs. baseline). Rates of ≥50 % improvement in NRS were 88.6 % at 1 month, 89.6 % at 6 months, 83.0 % at 12 months, and 59.5 % at 24 months. Side-lying tolerance improved from 21.5 % hips at baseline to 88.9 % reporting improvement at 1 month and 55.7 % at 24 months (p < 0.001). Sitting-to-standing ability showed sustained improvement at 81.0 % at 24 months. Composite responder rate declined from 88.6 % at 1 month to 57.0 % at 24 months. Subsequent invasive interventions were required in a minority of cases (2.5 % total hip arthroplasty; 11.4 % repeat TENEX).

**Conclusion:**

Combined PUT of the GMed and ITB provides durable pain relief and functional improvement in patients with refractory GTPS at two years post-procedure. Despite declining responder rates over time, this minimally invasive dual-target technique demonstrates a favorable safety profile and may delay or reduce the need for more invasive surgery. Further prospective studies are warranted to optimize patient selection and validate long-term efficacy.

## 1 Introduction

Greater Trochanteric Pain Syndrome (GTPS) is a common and often highly disabling condition characterized by chronic lateral hip pain. Symptoms are typically exacerbated by walking, climbing stairs, lying on the affected side, and rising from a seated position [1]. To help guide diagnosis of GTPS, physical examination maneuvers such as the FABER test (flexion, abduction, external rotation) and direct palpation of the lateral hip are commonly employed, alongside imaging modalities including X-ray radiography, magnetic resonance imaging (MRI), and ultrasound (US) [2]. Once considered primarily a bursitis, current literature suggests that GTPS is more accurately described as an enthesopathy of the hip, often related to gluteus medius (GMed) tendinopathy and iliotibial band (ITB) tightness [3].

Conservative medical management (CMM) remains the initial approach for GTPS, encompassing physical therapy, oral nonsteroidal anti-inflammatory medicines, or local corticosteroid injection (CSI) [4]. Platelet-rich plasma (PRP) injections are a promising and effective treatment, although not covered by insurance [5]. For patients unresponsive to CMM, surgical interventions such as ITB lengthening, trochanteric bursa debridement, and microtenotomy of the GMed can lead to long-term functional improvement [4].

However, invasive procedures are not suitable for all patients. For those diagnosed with GTPS who are poor surgical candidates or wish to avoid open procedures, percutaneous US-guided tenotomy (PUT) offers a less invasive alternative. Wahezi et al. demonstrated that ITB PUT significantly reduces pain and improves function at one-year follow-up in patients with GTPS, while Baker et al. reported favorable outcomes for GMed PUT in a similar patient population [6,7].

Building on these findings in GTPS, Wahezi et al. introduced a novel combined technique involving percutaneous TENEX (Tenex Health Inc., Lake Forest, CA, USA) releases of both the ITB and Gmed [8]. This dual-target strategy was based on growing evidence that both ITB and GMed pathology may contribute to the complex symptoms of GTPS, and that addressing both structures may enhance outcomes with minimal additional procedural risk. Early results at one year indicated that this dual-target approach provides effective symptom relief and functional gains [8]. Nevertheless, data on the long-term durability of this intervention remains limited. The present study addresses this gap by evaluating two-year follow-up outcomes in the same patient cohort, aiming to determine the sustained efficacy of ITB and GMed PUT.

## 2 Materials and methods

This retrospective study was approved by the Montefiore Health System institutional review board (IRB 2019–10877). Informed consent was waived due to the retrospective nature of the data collection. Patients who underwent the GMed/ITB tenotomy at the Montefiore Multidisciplinary Pain Center were identified via chart review between January 1, 2022, and August 31, 2023. The procedure was performed on consecutive patients, wherein all individuals meeting the inclusion criteria during the study period were enrolled in chronological order of presentation to minimize selection bias.

This study is a continuation of a previously published retrospective chart review reporting 12-month outcomes of patients who underwent ultrasound-guided GMed and ITB tenotomy for refractory GTPS [8]. The current investigation extends this cohort to include 24-month follow-up data from patients who could be contacted or whose chart contained complete information; specific sample size and baseline characteristics are presented in the Results section. Subject selection criteria for the TENEX procedure were consistent with those used in the prior publication and required patients to be over 18 years of age, demonstrate tendon hypo-echogenicity on the ITB and/or GMed tendons on ultrasound or edema on MRI, report an inability to tolerate side-lying for more than 5 min, experience lateral hip pain for more than six months that was reproducible on clinical palpation, and show failure to respond to conservative treatments such as rest, physical therapy, and oral medications. Patients who did not meet the subject selection criteria for the TENEX procedure, as well as those who underwent the procedure while pregnant or lactating, were not included in the study, to avoid potential confounding due to differences in pain management in this population. Patients who underwent another invasive procedure in the same hip following the initial TENEX procedure were not included in the dataset beyond that point; however, the occurrence of these interventions was documented for completeness.

The current study builds upon that cohort by analyzing follow-up data from 79 hips for which 2-year data were available, or patients could be contacted by phone. For the 79 hips included in the present study, a retrospective chart review also provided data from earlier time points. Data for the 2-year outcomes were obtained through structured phone interviews, while demographic and baseline clinical information were extracted from medical records.

The primary outcome was improvement in hip pain, assessed using the Numeric Rating Scale (NRS). Pain improvement was defined as a reduction of 50 % or more compared to baseline. Secondary outcomes included side-lying tolerance, sitting-to-standing tolerance, responder rate and the need for additional interventional or surgical procedures targeting the affected hip after the tenotomy. Side-lying tolerance was defined as the ability to comfortably lie on the side for as long as the patient could tolerate their contralateral (unaffected) hip, or, in cases of bilateral involvement, for as long as the patient could lie on the affected hip before symptoms began, without significant pain, discomfort, or needing to change position. Improvement was defined as the patient being able to maintain uninterrupted side-lying tolerance throughout the night; patients who were unable to do so were considered not improved.

Sitting-to-standing tolerance referred to the ability to stand up and walk without difficulty after sitting for 30 min. Improvement was defined as the absence of pain or stiffness interfering with this transition; patients who reported difficulty standing or walking immediately after sitting, or who needed to pause before walking, were considered not improved.

Patients were considered responders if they met both of the following criteria: a ≥50 % reduction in hip pain and full-lying tolerance through the night. This dual criterion was selected because pain and difficulty lying on the affected side represent the two most prominent symptoms experienced by patients with GTPS [8].

In addition to the primary and secondary outcomes, data was collected regarding any subsequent invasive procedures performed on the same side following the GMed/ITB TENEX procedure, including the type and timing of such interventions.

Descriptive statistics were used to summarize patient demographics and clinical outcomes. Continuous variables were presented as means with standard deviations (SD), as medians with interquartile ranges (IQR), and categorical variables were reported as frequencies and percentages. Paired t-tests were used to evaluate changes in continuous outcomes over time. A p-value <0.05 was considered statistically significant.

## 3 Results

At baseline, a thorough chart review identified 115 hips from 95 patients diagnosed with GTPS, including 75 patients with unilateral and 20 with bilateral hip involvement. In that previously published study, follow-up data were successfully collected for 68 hips, with the remaining cases excluded due to missing data or incomplete 12-month follow-up [8].

In the present study, 79 hips from 69 patients (10 with bilateral and 59 with unilateral involvement) had available 24-month follow-up data. All 79 hips had data at 1- and 24-month follow-up, 77 hips had data at 6 months, and 53 hips had data at 12 months.

The mean age of 69 patients was 63.17 years, with a mean BMI of 32.75. Of the 69 patients, 3 were male and 66 were female. Mean ITB thickness was 6.6 mm. The average follow-up duration was 30.29 months (range 21–40 months) (see Table 1).

**Table 1:** 

Study Demographics	
Number of patients	69
Number of hips treated	79
Male	3
Female	66
Age ± SD	63.17 (±11.34)
BMI ± SD	32.74 (±7.26)
Laterality	39L/40R
ITB Thickness	6.6
FU Duration	30.29 M (Range: 21–40)

∗Total hips at 1 month 79, at 6 month 77, at 12 month 53, at 24 month 79.

Baseline pain scores showed a median of 10 (IQR 9–10). Post-procedure, there was a significant improvement in pain and tolerance to weight-bearing and side-lying positions. Post-procedure, median NRS significantly decreased to 2 (IQR 1–4) at 1, 6, and 12 months, then increased to 6 (IQR 4–10) at 24 months, although still significantly improved compared to baseline (p < 0.001). Rates of ≥50 % improvement in NRS were 88.6 % at 1 month, 89.6 % at 6 months, 83.02 % at 12 months, and 59.5 % at 24 months (see Table 2).

**Table 2:** 

	NRS		Standing tolerance		Side lying tolerance	
Pre-procedure	10 (IQRs 9,10)		∗		∗∗	
Post-procedure 1 month	2 (IQRs 1,4)	p < 0.001	87.34 %	p < 0.001	89.87 %	p < 0.001
Post-procedure 6 month	2 (IQRs 1,4)	p < 0.001	90.91 %	p < 0.001	89.61 %	p < 0.001
Post-procedure 12 month	2 (IQRs 1,4)	p < 0.001	84.91 %	p < 0.001	83.02 %	p < 0.001
Post-procedure 24 month	6 (IQRs 4,10)	p < 0.001	81.01 %	p < 0.001	55.70 %	p < 0.001

∗In the pre-procedure period, only 13 out of 69 patients were able to stand still for a while and walk from sitting.

∗∗In the pre-procedure period, only 17 out of 69 patients were able to lie on their side for 5–15 min.

Similarly, side-lying tolerance improved significantly. At baseline, most subjects could not lie on their side without significant pain, discomfort, or needing to change position, and only 17 of 79 subjects (21.5 %) could do so. At 1-month post-procedure, 88.87 % reported improvement, which remained relatively stable through 12 months. At 24 months, the improvement rate declined to 55.7 % but remained statistically significant (p < 0.001).

At baseline, only 13 out of 75 subjects could tolerate sitting-to-stand, while the remaining subjects were nearly unable to do. Following the procedure, sitting-to-standing improved significantly, with 83.34 % of subjects reporting improvement at 1 month, 90.91 % at 6 months, 84.91 % at 12 months, and 81.01 % at 24 months.

Responder rate, defined as a ≥50 % reduction in hip pain and full-lying tolerance through the night, was 88.60 % at 1 month, 89.61 % at 6 months, 79.24 % at 12 months, and 56.96 % at 24 months.

Following the initial TENEX procedure, 10 subjects (12.7 %) required subsequent invasive interventions. Of the 79 hips, 9 (11.4 %) required a repeat TENEX procedure, 1 (1.3 %) underwent arthroscopic ITB release 15 months after the initial TENEX.

For the 9 hips that underwent repeat TENEX, the mean interval after the initial procedure was 23.6 months (range, 18–32 month), with a mean follow-up of 31.9 months after the initial TENEX and 10.3 months after the repeat procedure. The baseline median pain score was 10 (IQR, 9–10), which decreased to 8 (IQR, 7–10) at the 2-year follow-up (prior to the repeat TENEX), remaining significantly higher than the overall cohort's 2-year NRS (p < 0.005). None of these subjects were able to lie on their side at baseline; at the 2-year follow-up, side-lying improvement was observed in 22.2 % and sit-to-stand improvement in 44.4 %, with an overall responder rate of 22.2 %. No significant differences in age, body mass index (BMI), or ITB thickness were observed between this subgroup and the overall cohort (ITB thickness: 7.33 mm in repeat TENEX group vs. 6.6 mm in full cohort, p = 0.33).

Additionally, 6 hips (7.6 %) received a CSI targeting the GT and/or ITB regions after the initial TENEX procedure, with a mean interval of 16 months. The median pain score decreased from 9 (IQR, 8–10) at baseline to 7 (IQR, 4–9) at the 2-year follow-up. No other significant differences were noted between this group and the overall cohort.

## 4 Discussion

This long-term follow-up study of combined PUT of the GMed and ITB in patients with recalcitrant GTPS demonstrates sustained, clinically meaningful improvements in pain and function. While short-term outcomes were previously reported as favorable at 12 months, this extended analysis confirms that a substantial proportion of patients continue to benefit in the long term, suggesting that this minimally invasive intervention may provide durable symptom relief in appropriately selected patients [6].

GTPS is increasingly recognized as a chronic degenerative enthesopathy rather than an isolated bursitis, with key pathologic contributions from GMed and minimus tendinopathy, ITB tension, and surrounding fascial pathology [9,10]. This mechanistic understanding has shifted treatment paradigms from simple anti-inflammatory strategies toward interventions targeting structural pathology. In this study, the dual-target approach was associated with symptomatic improvement in patients with GTPS, suggesting that concurrently addressing the gluteal and ITB regions may be beneficial in managing this condition. Moreover, the consistent response to the dual-target PUT in this cohort highlights the potential relevance of considering multiple pain generators in GTPS rather than focusing on a single pathological source. These findings support the hypothesis that treating both the gluteal and ITB structures may provide more effective symptom relief by targeting distinct yet interrelated sources of pain.

Many patients in this cohort presented high baseline pain scores, with a median NRS of 10, likely because these individuals had already undergone multiple failed prior treatments, making them appropriate candidates for the TENEX procedure. Despite this high baseline severity, long-term post-procedure outcomes were favorable: nearly 60 % of patients continued to experience ≥50 % pain reduction, and 57 % met the composite responder definition. This aligns with trends seen in other studies of tendon-focused interventions. PUT, first developed for common extensor tendinopathy, has since been shown to yield good outcomes for various tendinopathies, including the Gmed [7,11,12]. Baker et al. reported promising results in a pilot study of GMed PUT in the hip, with most patients showing pain and functional improvement over several months [7]. The current study builds on that evidence by applying combined GMed and ITB PUT technique and extending the follow-up to an average of two years.

The durability of response observed here compares favorably with that of other non-surgical treatments. While widely used, CSIs typically provide only short-term benefits, with high recurrence rates within 3–6 months [13,14]. PRP has emerged as an alternative option to steroid, with some studies suggesting greater durability than steroids [15]. However, its benefit beyond one year remains inconsistent, and cost/access barriers persist. In contrast, PUT is routinely performed as a single-session, outpatient intervention, with low complication rates and a favorable risk-benefit profile. Although a small subset of patients in this cohort required repeat procedures, the treatment is generally intended as a one-time intervention and provides durable symptom relief.

Our low revision and surgical conversion rates are also noteworthy. Only one subject (1.3 %) underwent arthroscopic ITB release, and 9 (11.4 %) required repeat tenotomy. These findings suggest that PUT may serve as a valuable option for delaying or avoiding more invasive procedures in patients with refractory GTPS. Prior work has shown that surgery for GTPS, such as GMed repair or ITB release, carries higher risks and longer recovery times, despite often yielding good outcomes in selected patients [16].

Nevertheless, reducing response rates over the long term raises critical clinical considerations. While some decline is expected due to the chronic and often progressive nature of tendinopathy, it may also reflect patient-specific factors such as mechanical overuse, hip biomechanics, and comorbid spinal or sacroiliac pathology.

Its retrospective design and single-center setting limit this study. While phone follow-up allowed us to capture extended data, self-reported outcomes may be subject to recall bias. Additionally, although our dual-targeted PUT strategy is promising, there is no randomized comparison to GMed- or ITB-only approaches. Future prospective trials comparing single-vs. dual-site tenotomy are warranted to clarify the additive benefit of the combined intervention and identify patient-level predictors of sustained improvement.

## 5 Conclusion

The combined PUT of the GMed and ITB offers durable pain relief and functional improvement for patients with recalcitrant GTPS.

## Funding sources

This research did not receive any specific grant from funding agencies in the public, commercial, or non-profit sectors.

## Conflict of interest

All authors declared "no conflict of interest" for this publication.

## Data Availability

All data generated and analyzed during this study are included in this published article.
